# Supplementary material for: Transcriptional signals of transformation in human cancer
Source: Genome Med. 2024 Jan 9;16:8. doi: 10.1186/s13073-023-01279-z (PMC10775554; doi:10.1186/s13073-023-01279-z)
Supplement: Supplementary file 2 — Additional file 2: Fig. S1. Relationship between stemness and early embryo scores. Fig. S2. Cell signal analysis of all TCGA samples. Fig. S3. Cell signal analysis of all TARGET samples. Fig. S4. Cell signal analysis of all StJudes samples. Fig. S5. Cell signal analysis of all liver samples. Fig. S6. Cell signal analysis of all gut samples. Fig. S7. Survival prediction for TCGA and TARGET cancers [file 13073_2023_1279_MOESM2_ESM.pdf]

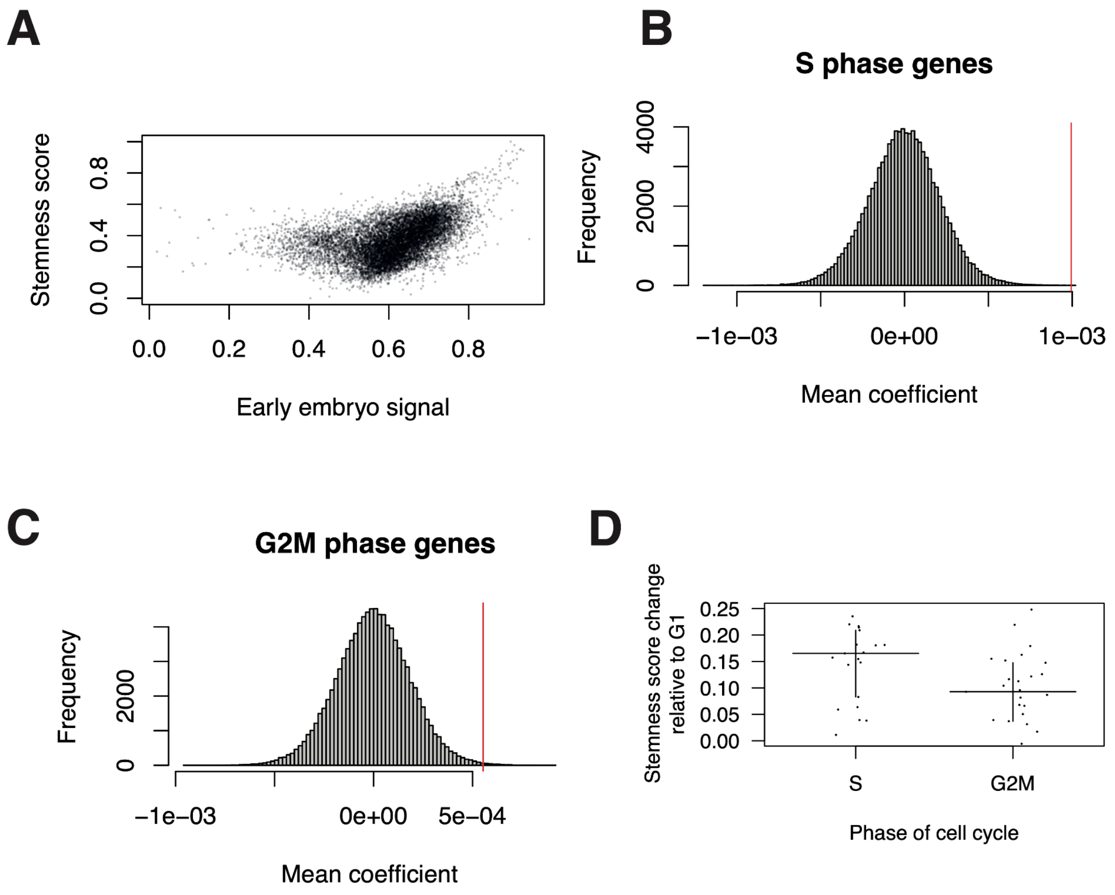

Fig. S1 - Relationship between stemness and early embryo scores

A. **Correlation between stemness and early embryo scores:** Stemness score (y-axis) as a function of early embryo signal (x-axis), for bulk cancer transcriptomes (dots).

B. **Significance of S phase genes in stemness score:** The distribution across many randomly selected gene sets (y-axis) of average coefficients for that gene set in determining the stemness score (x-axis). Values further away from zero mean that a set of genes has a consistent contribution to determining the stemness value. The value corresponding to the set of genes linked to S-phase of the cell cycle is marked with a red line.

C. **Significance of G2M phase genes in stemness score:** The same as **B.** but using genes associated with G2 and M phase of the cell cycle.

D. **Shift in stemness score with cell cycle phase:** The single cell fetal liver reference data was used to construct pseudo-bulk counts from which stemness scores were calculated. For each cell type, a separate pseudo-bulk was constructed for cells in each phase of the cell cycle. The difference in stemness score for each cell type (y-axis) was calculated between other phases of the cell cycle (x-axis) and G1 phase. Horizontal lines indicate the median shift across all cell types, while the vertical lines indicate the 1st and 3rd quartiles.

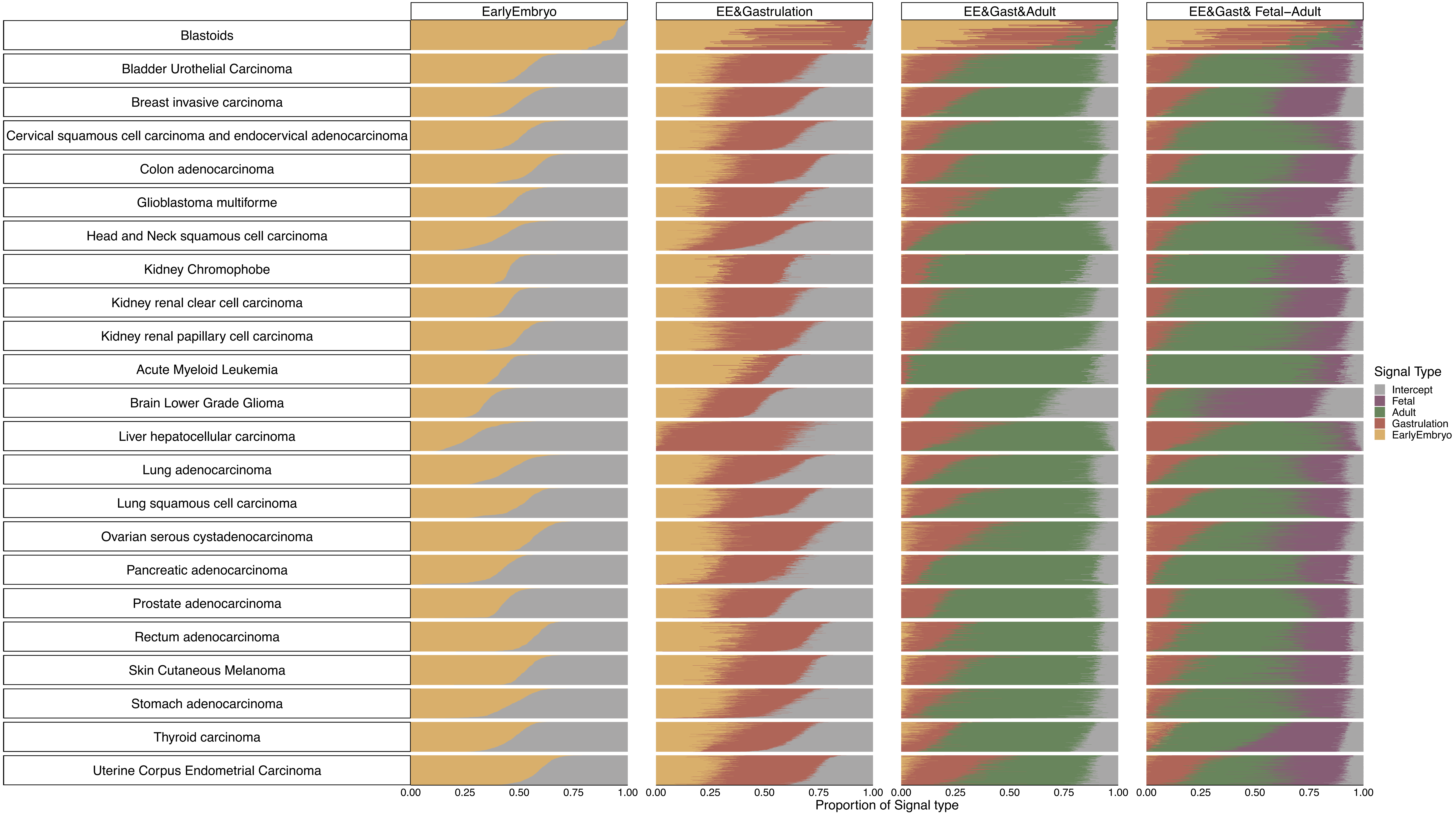
EarlyEmbryo
EE&Gastrulation
EE&Gast&Adult
EE&Gast& Fetal−Adult
Blastoids
Bladder Urothelial Carcinoma
Breast invasive carcinoma
Cervical squamous cell carcinoma and endocervical adenocarcinoma
Colon adenocarcinoma
Glioblastoma multiforme
Head and Neck squamous cell carcinoma
Kidney Chromophobe
Kidney renal clear cell carcinoma
Kidney renal papillary cell carcinoma
Acute Myeloid Leukemia
Brain Lower Grade Glioma
Liver hepatocellular carcinoma
Lung adenocarcinoma
Lung squamous cell carcinoma
Ovarian serous cystadenocarcinoma
Pancreatic adenocarcinoma
Prostate adenocarcinoma
Rectum adenocarcinoma
Skin Cutaneous Melanoma
Stomach adenocarcinoma
Thyroid carcinoma
Uterine Corpus Endometrial Carcinoma
0.00
0.25
0.50
0.75
1.00
Proportion of Signal type
Signal Type
Intercept
Fetal
Adult
Gastrulation
EarlyEmbryo

Fig. S2 - Cell signal analysis of all TCGA samples

Relative contribution of single cell reference populations (x-axis) in explaining all TCGA bulk adult cancer transcriptomes (y-axis) using cell signal analysis for different combinations of reference populations (columns splits, with heading indicating reference combination). Individual cancer transcriptomes (rows) are split into groups based on cancer types, as indicated by the row split titles. Colours indicate the contribution to each transcriptome of each reference type, as described by the legend on the right.

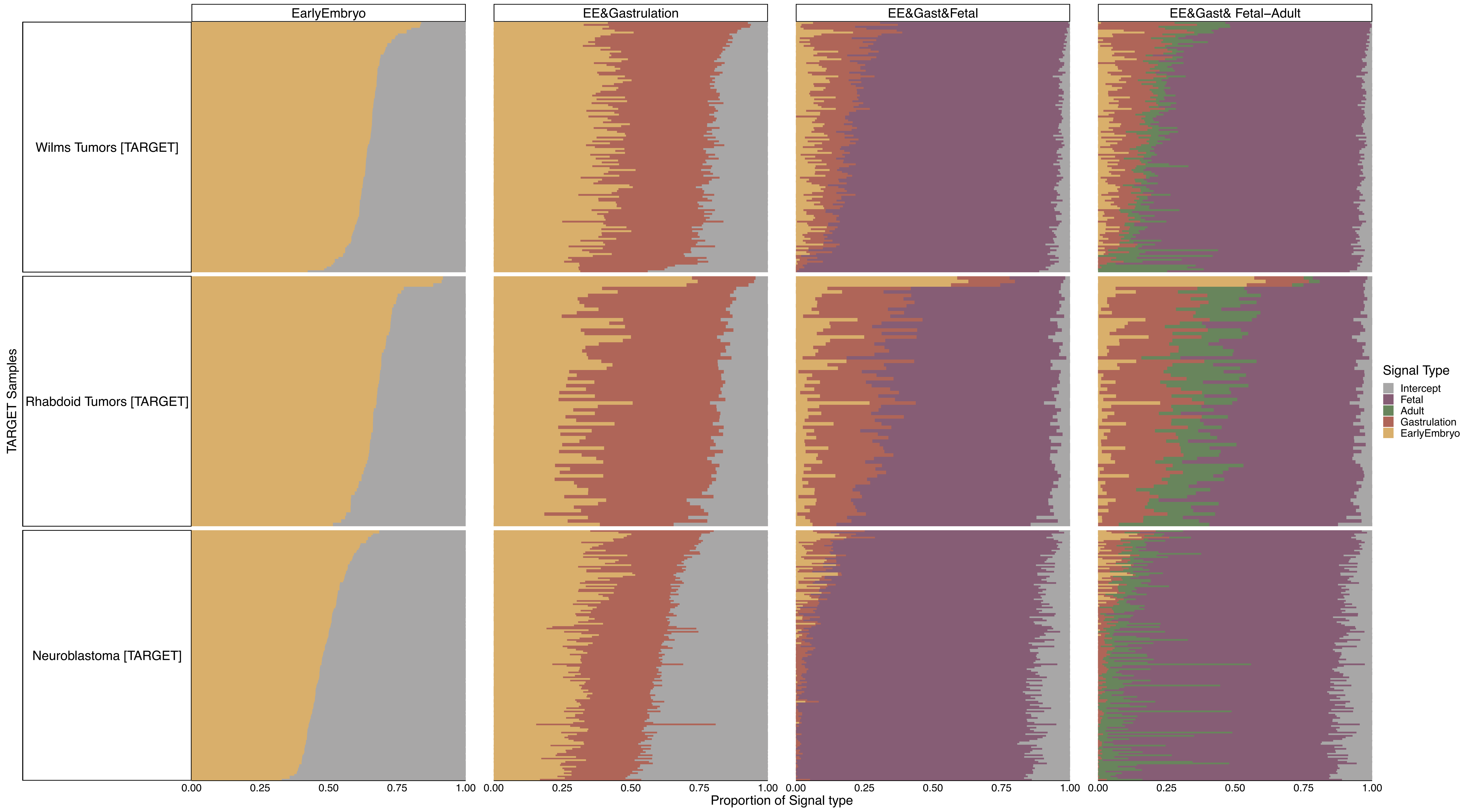

EarlyEmbryo
EE&Gastrulation
EE&Gast&Fetal
EE&Gast& Fetal−Adult
Wilms Tumors [TARGET]
Rhabdoid Tumors [TARGET]
Neuroblastoma [TARGET]
TARGET Samples
0.00 0.25 0.50 0.75 1.00
Proportion of Signal type
Signal Type
Intercept
Fetal
Adult
Gastrulation
EarlyEmbryo

Fig. S3 - Cell signal analysis of all TARGET samples

Relative contribution of single cell reference populations (x-axis) in explaining all TARGET bulk childhood cancer transcriptomes (y-axis) using cell signal analysis for different combinations of reference populations (columns splits, with heading indicating reference combination). Individual cancer transcriptomes (rows) are split into groups based on cancer types, as indicated by the row split titles. Colours indicate the contribution to each transcriptome of each reference type, as described by the legend on the right.

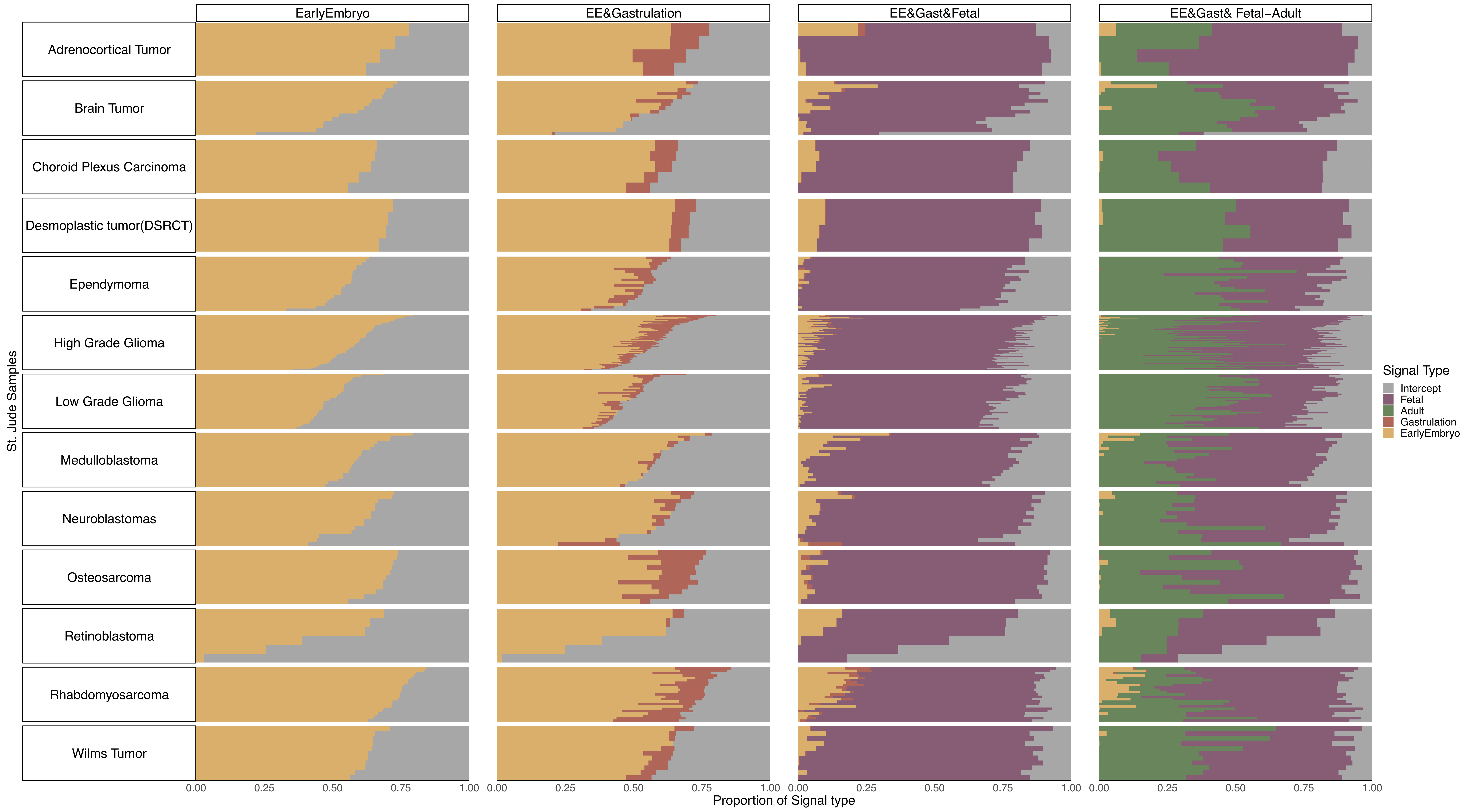
EarlyEmbryo
EE&Gastrulation
EE&Gast&Fetal
EE&Gast& Fetal–Adult
Adrenocortical Tumor
Brain Tumor
Choroid Plexus Carcinoma
Desmoplastic tumor(DSRCT)
Ependymoma
High Grade Glioma
Low Grade Glioma
Medulloblastoma
Neuroblastomas
Osteosarcoma
Retinoblastoma
Rhabdomyosarcoma
Wilms Tumor
St. Jude Samples
0.00
0.25
0.50
0.75
1.00
Proportion of Signal type
Signal Type
Intercept
Fetal
Adult
Gastrulation
EarlyEmbryo

Fig. S4 - Cell signal analysis of all StJudes samples

Relative contribution of single cell reference populations (x-axis) in explaining all StJudes bulk childhood cancer transcriptomes (y-axis) using cell signal analysis for different combinations of reference populations (columns splits, with heading indicating reference combination). Individual cancer transcriptomes (rows) are split into groups based on cancer types, as indicated by the row split titles. Colours indicate the contribution to each transcriptome of each reference type, as described by the legend on the right.

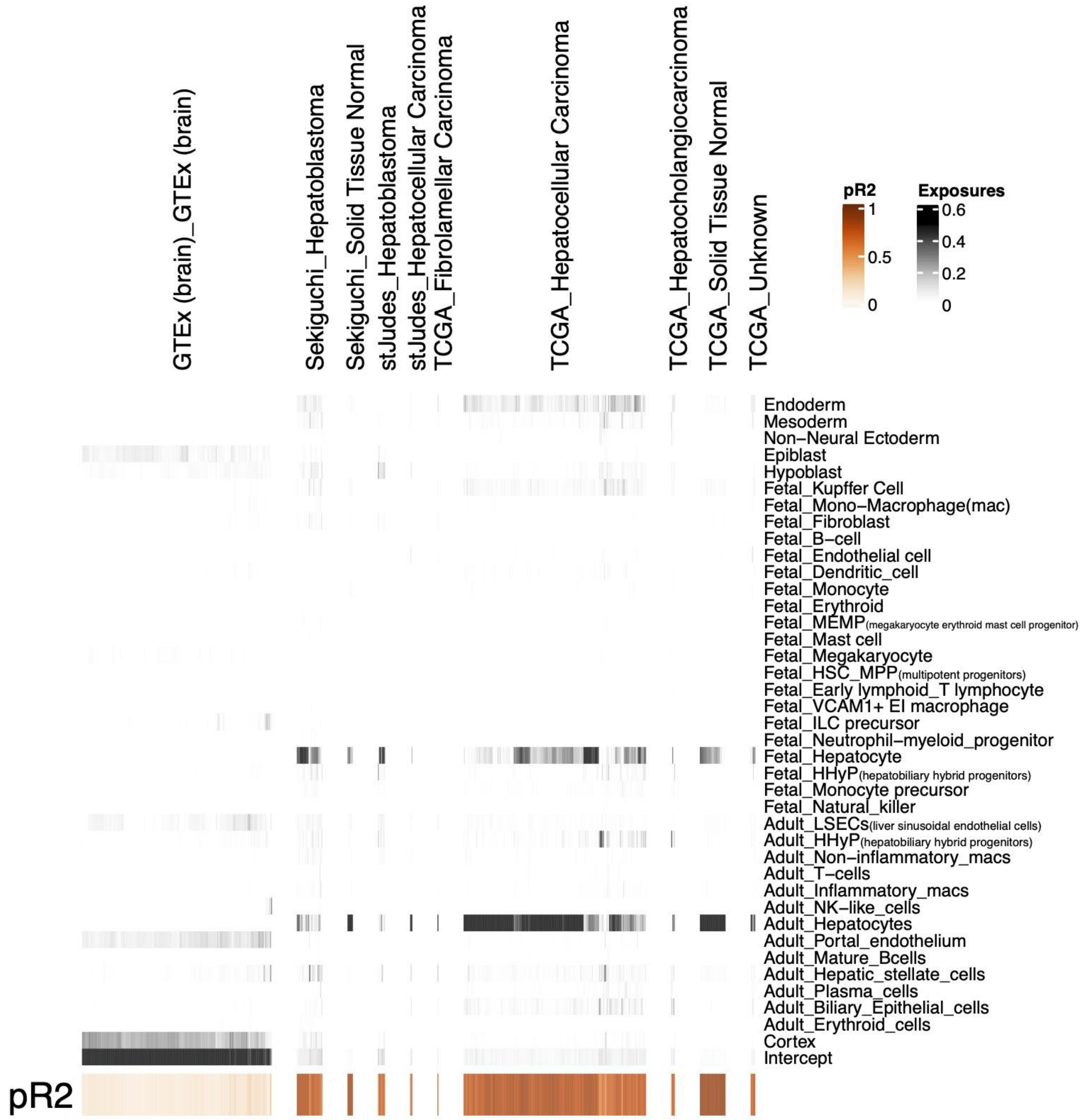

Fig. S5 - Cell signal analysis of all liver samples

Relative contribution of single cell populations from the fetal and adult liver, gastrulation, and early embryo (y-axis) in explaining bulk transcriptomes (y-axis) using cell signal analysis. Bulk transcriptomes (columns) are grouped together into the categories indicated by the top labels and the relative contribution in explaining the signal is indicated by the intensity of the greyscale as indicated by the legend. For each bulk transcriptome, a goodness of fit (pseudo R squared) was calculated and is indicated by the intensity of the orange colour in the bottom row, as described by the legend.

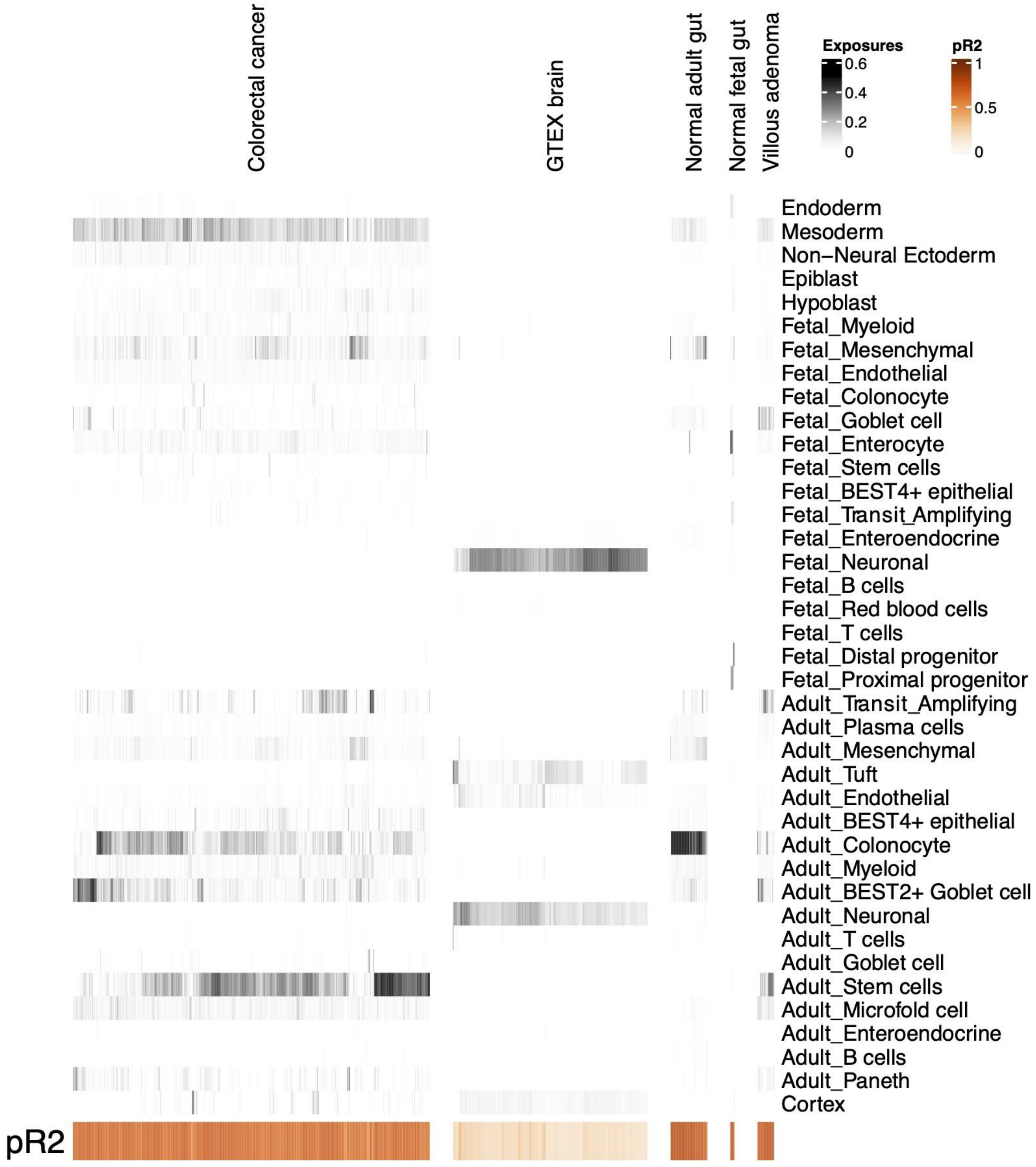

Fig. S6 - Cell signal analysis of all gut samples

Relative contribution of single cell populations from the fetal and adult gut, gastrulation, and early embryo (y-axis) in explaining bulk transcriptomes (y-axis) using cell signal analysis. Bulk transcriptomes (columns) are grouped together into the categories indicated by the top labels and the relative contribution in explaining the signal is indicated by the intensity of the greyscale as indicated by the legend. For each bulk transcriptome, a goodness of fit (pseudo R squared) was calculated and is indicated by the intensity of the orange colour in the bottom row, as described by the legend.

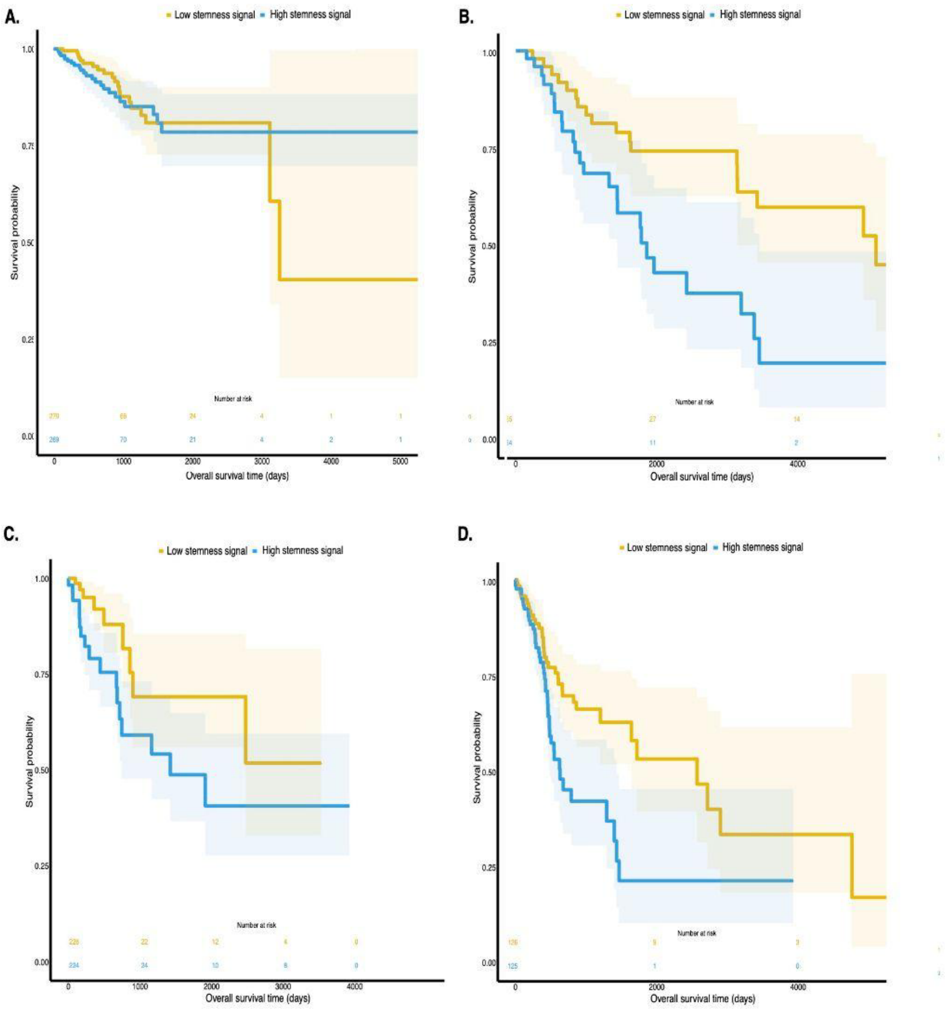

Fig. S7 - Survival prediction for TCGA and TARGET cancers

Kaplan–Meier curves showing survival for first and fourth quartile of dedifferentiation fraction($p<0.05$, exact p-values and results are deposited in TableS3) suggesting tumour samples with high stemness signal have worse survival outcome. A. TARGET NBL, B. TCGA-SKCM C. TCGA-COAD D. TCGA-HNSC
